# Core Competencies for Serious Illness Conversations: An Integrative Systematic Review

**DOI:** 10.1177/08258597241245022

**Published:** 2024-04-01

**Authors:** Susanna Pusa, Rebecca Baxter, Sofia Andersson, Erik K. Fromme, Joanna Paladino, Anna Sandgren

**Affiliations:** 1 Center for Collaborative Palliative Care, Department of Health and Caring Sciences, 4180 Linnaeus University, Växjö, Sweden; 2 Department of Nursing, Umeå University, Umeå, Sweden; 3 480938 Ariadne Labs, Boston, Massachusetts, USA; 4 1811 Harvard Medical School, Boston, Massachusetts, USA; 5 1855 Dana-Farber Cancer Institute, Boston, Massachusetts, USA; 6 2348 Massachusetts General Hospital, Boston, Massachusetts, USA

**Keywords:** clinical competence, health communication, palliative care, serious illness conversations, serious illness care program, systematic review

## Abstract

**Objective:** The Serious Illness Care Program was developed to support goals and values discussions between seriously ill patients and their clinicians. The *core competencies*, that is, the essential clinical conversation skills that are described as requisite for effective serious illness conversations (SICs) in practice, have not yet been explicated. This integrative systematic review aimed to identify core competencies for SICs in the context of the Serious Illness Care Program. **Methods:** Articles published between January 2014 and March 2023 were identified in MEDLINE, PsycINFO, CINAHL, and PubMed databases. In total, 313 records underwent title and abstract screening, and 96 full-text articles were assessed for eligibility. The articles were critically appraised using the Joanna Briggs Institute Critical Appraisal Guidelines, and data were analyzed using thematic synthesis. **Results:** In total, 53 articles were included. Clinicians’ core competencies for SICs were described in 3 themes: conversation resources, intrapersonal capabilities, and interpersonal capabilities. Conversation resources included using the conversation guide as a tool, together with applying appropriate communication skills to support better communication. Intrapersonal capabilities included calibrating one's own attitudes and mindset as well as confidence and self-assurance to engage in SICs. Interpersonal capabilities focused on the clinician's ability to interact with patients and family members to foster a mutually trusting relationship, including empathetic communication with attention and adherence to patient and family members views, goals, needs, and preferences. **Conclusions:** Clinicians need to efficiently combine conversation resources with intrapersonal and interpersonal skills to successfully conduct and interact in SICs.

## Introduction

Effective and empathetic communication is a core competency for healthcare professionals, perhaps none more so than for those working with seriously ill patients. It is argued that competence to hold serious illness conversations (SICs) can be taught, learnt, and applied in clinical practice.^1,2^ Despite myriad studies affirming the importance of developing clinical conversation skills, clinicians have described lacking confidence when it comes to talking to patients about emotional or sensitive topics and feeling uncertain about how to develop competence in discussing such matters.^3,4^ This can result in clinicians avoiding difficult conversations altogether, thereby restricting opportunities for the provision of person-centered and goal-concordant care.^4,5^

The Serious Illness Care Program (SICP), inclusive of the Serious Illness Conversation Guide (SICG), was developed by Ariadne Labs (Boston, Massachusetts, USA) to augment discussions around seriously ill patients’ values, goals, preferences, and priorities.^6^ The SICP includes structured tools, training, and technical support for program implementation at clinical and organizational levels.^6^ The SICP aims to promote more, better, and earlier conversations between clinicians and patients with serious illness^4^ and can be undertaken using different formats, including in-person, over the telephone, and using digital mediums.^6^ The SICP was initially designed and studied for patients in oncology care and is now being used among diverse patient groups, clinical settings, and professional user groups.^7,8^ This is encouraging as more patients and family/caregivers in need of SIC have access to them; however, this also poses a challenge for clinicians as it is unclear what essential competencies are needed to use the SICP and SICG to accomplish successful SIC. Competence as a concept is complex to define and several variants exist.^9^ It has been described as a multifaceted concept covering more than knowledge acquisition alone, since it includes the understanding and application of knowledge, clinical skills, interpersonal skills, problem-solving, clinical judgment, and technical skills.^10^ For this study, core competencies are defined as a mixture of attributes comprising applied knowledge, skills, and attitudes^11^ that qualify a clinician to perform SIC competently.

The SICP includes clinician training to build confidence and self-efficacy in conducting SIC. Studies exploring the effect of the SICP have found that targeted SIC resulted in more goal-concordant discussions and improved experiences of palliative care provision for healthcare professionals.^12^ While these advancements are promising, there continues to be confusion surrounding the core competencies, that is, the *essential* clinical conversation attributes that are required to have SIC. Generating a clear outline of the core competencies for SIC in the context of the SICP is critical to inform current and future training approaches, learning strategies, and clinical requisites. This integrative systematic review aimed to identify core competencies for SICs in the context of the SICP.

## Methods

### Study Design

To ensure methodological rigor and minimize the risk of bias this integrative systematic review was conducted as per the guidelines set out by The Joanna Briggs Institute (JBI) and has been reported using the Preferred Reporting Items for Systematic Reviews and Meta-Analyses Checklist.^13^ This study was not registered and the protocol has not been published.

### Search Strategy

The bibliographic databases MEDLINE, PsycINFO, CINAHL, and PubMed were searched on the 20th of March 2023. The search terms were developed in collaboration with a university librarian and included: “serious illness program*” OR “serious illness care” OR “serious illness conversation*” OR “serious illness model” OR “serious illness communication.” The full search terms and limiters for each database are provided in Supplemental Material A. The reference lists of the included studies were hand-searched to ensure the completeness of the review. Additionally, Ariadne Labs provided a list of known publications (*n* = 44) related to the SICP and/or SICG. The sensitivity of the search strategy was confirmed through the identification of seminal publications from the SICP, SICG, and Ariadne Labs in the search results.

### Eligibility Criteria

To collect data relevant to the study aim, description/s of core competencies for SIC were sought in articles pertaining to the SICP and/or SICG. Inclusion criteria were developed to ensure that the articles contained information relevant to the study aim (Table 1). As the SICP was established following a literature review from 2014, the search was restricted to articles published between 1 January 2014 and 20 March 2023. If articles were not connected to Ariadne Labs’ SICP/SICG or did not explicitly describe at least one competence related to conducting SIC, they were excluded. Conference papers and letters to the editor were not eligible for inclusion.

**Table 1.** Inclusion Criteria.

Inclusion criteria
(1) Publication date: 1 January 2014 to 20 March 2023
(2) Publication language: English
(3) Related to Ariadne Labs’ SICP and/or SICG
(4) Meaningful description of (at least one) competency for serious illness conversations

SICP = Serious Illness Care Program; SICG = Serious Illness Care Guide.

### Selection Process

Following the removal of duplicate publications, the first author (SP) scanned all titles, abstracts, keywords, and, if required, the full-text article, to identify those that met the inclusion criteria. Two authors (SP and RB) then screened all full-text articles against the inclusion criteria. Any disagreements were resolved through discussion until a consensus was reached.

### Data Collection

Original source data from the methods, results, discussion, and/or conclusions sections of the included articles were eligible for extraction. Original source data were text that was presented as findings, descriptions, interpretations, or ideas written by the author/s of the paper. A data extraction template was developed to gather general information about the article’s characteristics, including the aim, context, study design, and affiliation with the SICP/SICG. A further extraction template was used to organize data describing SIC competencies. To calibrate this process, data from a random sample of six articles were extracted. The second author (RB) then extracted data from all articles, which was then verified by the first author (SP) for consistency and uniformity. Any disagreements were resolved by discussion and reappraisal.

### Critical Appraisal

All articles were evaluated by the first author (SP) to assess methodological quality and risk of bias as per the JBI critical appraisal checklists.^14^ The checklist that was most consistent with the study design was selected to evaluate the included articles (ie, cross-sectional, case-control, qualitative, etc). As JBI does not yet offer a checklist for mixed methods studies they provided advice via email that multiple checklists should be completed for studies reporting more than one method. Each article was assessed by answering “yes,” “no,” “unclear,” or “not applicable” to the checklist criteria. A random sample (approximately 20%) of the studies were reviewed by RB for comparative evaluation. Any inconsistencies between the reviewers (eg, selection of checklist, quality assessment criteria) were resolved through discussion. As different checklists were used for different study designs the quality assessments were not used to exclude articles but were instead used to provide information regarding the quality and comparability of the included articles to better evaluate their content.

### Data Analysis

Thematic synthesis analysis was selected for this study as it allows for the identification and aggregation of patterns across diverse textual sources.^15,16^ As described by Thomas and Harden,^15^ this method comprises 3 stages: free coding, thematic organization, and analytic thematic development. First, textual data were inductively explored for descriptions of SIC competencies and coded accordingly. Next, similar codes were grouped and descriptive subthemes were formulated. Finally, analytical themes were constructed to provide novel interpretations that extended beyond surface-level descriptions to generate new insights from the existing literature. The preliminary themes were discussed among the author group and all authors agreed upon the final results.

## Results

### Study Selection

**Figure 1.**
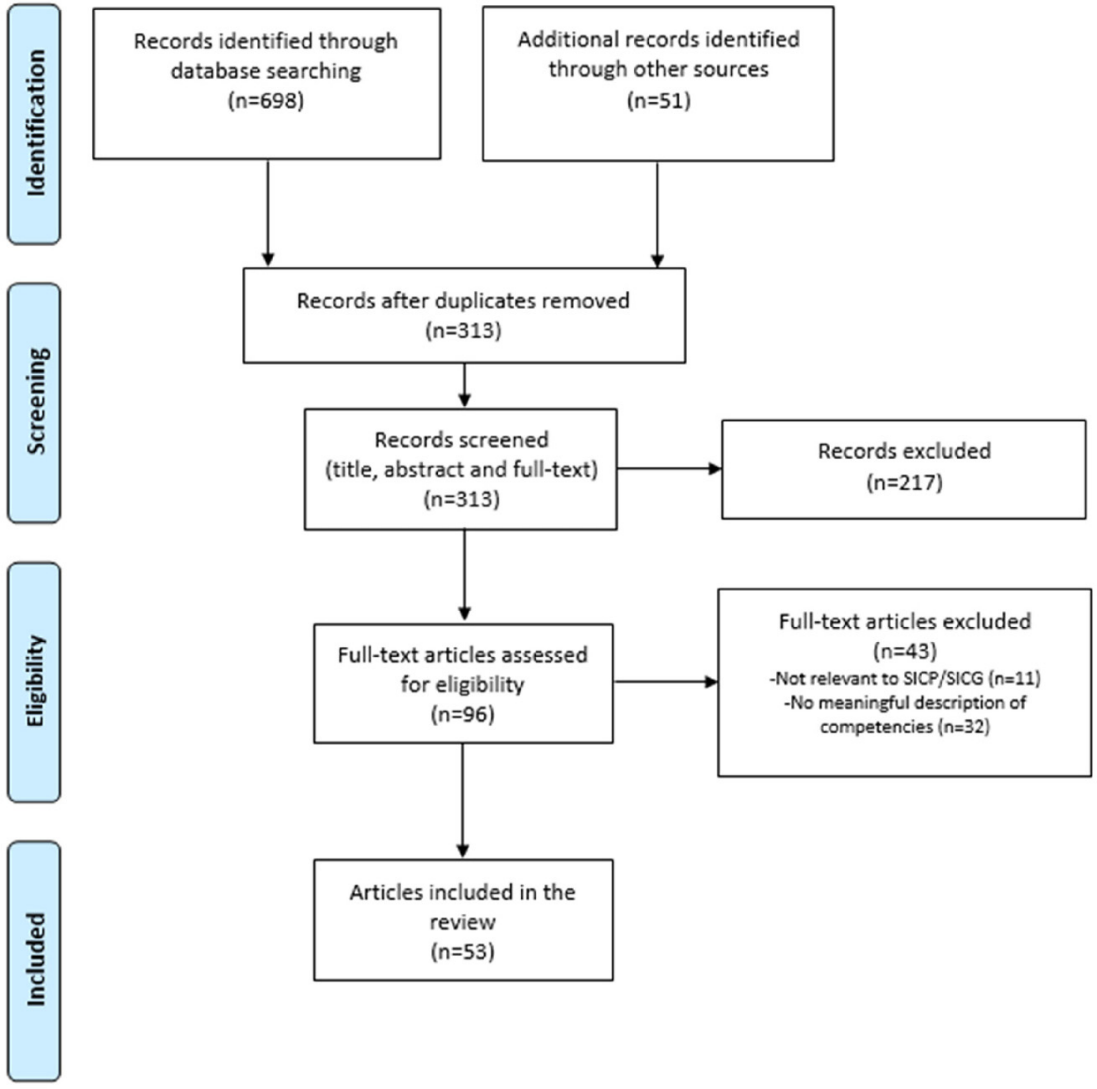
PRISMA flow diagram.

The search revealed 698 studies. Fifty-one additional studies were located through the reference list review (*n* = 7) and the list of articles provided by Ariadne Labs (*n* = 44). Of these 749 articles, 436 were duplicates. In total, 53 articles met the inclusion criteria (Figure 1).

PRISMA, Preferred Reporting Items for Systematic Reviews and Meta-Analyses.

### Study Characteristics

The majority of the included articles originated from the United States (*n* = 40) and Canada (*n* = 10). The remaining articles were from the United Kingdom (*n* = 1), Sweden (*n* = 1), and Denmark (*n* = 1). Of the included articles, 13 used qualitative methods, 15 used some form of mixed methods, and 16 used quantitative methods. Nine articles were categorized using the JBI criteria as text and opinion articles. Detailed JBI critical appraisal checklist responses are presented in online Supplemental Material B. The list of included articles and a summary of their characteristics are presented in Table 2.

**Table 2.** Summary of Included Articles.

Author/s, Year	Clinical context	Clinicians/users	Country	Critical appraisal checklist/s
Andersson et al, 2022^17^	Acute care hospitals	Physicians	Sweden	Qualitative research
Baran et al, 2019^18^	Primary care	Primary care clinicians	USA	Text and opinion
Beddard-Huber et al, 2021^19^	General	Physicians, nurse practitioners, nurses, allied health	Canada	Text and opinion
Bernacki et al, 2015^20^	Oncology	Nurse practitioners, physician assistants	USA	Text and opinion
Borregaard Myrhøj et al, 2022^21^	Multiple myeloma	Physicians, nurses	Denmark	Qualitative research
Daly et al, 2022^22^	Family medicine	Physicians, nurses, other clinical staff	USA	Analytical cross-sectional studies
Daubman et al, 2020^23^	Multiple contexts	Clinicians—not specified	USA	Analytical cross-sectional studies
DeCourcey et al, 2021^24^	Pediatrics	Physicians, nurse practitioners, nurses, psychosocial clinicians	USA	Qualitative research
Gace et al, 2020^25^	General medical inpatient	Physicians, nurse practitioners, physician assistants, nurses, social workers	USA	Cohort studies
Geerse et al, 2019^26^	Oncology	Physicians, nurse practitioners, physician assistants	USA	Qualitative research
Geerse et al, 2021^27^	Oncology	Physicians, nurse practioners, physician assistants	USA	Analytical cross-sectional studies and qualitative research
Greenwald et al, 2020^28^	General medical inpatient	Physicians, nurse practitioners, physician assistants	USA	Cohort studies
Greenwald et al, 2021^29^	Hospital setting	Clinicians—not specified	USA	Quasi-experimental studies
Hafid et al, 2021^30^	Primary care	Physicians, residents, nurse practitioners, nurses, social workers	Canada	Qualitative research and quasi-experimental studies
Jain et al, 2020^31^	Not stated	Clinicians—not specified	USA	Text and opinion
Jacobsen et al, 2022^32^	Not stated	Clinicians—not specified	USA	Text and opinion
Karim et al, 2022^33^	Oncology	Physicians, physician assistants, nurse practitioners	USA	Text and opinion
King et al, 2022^34^	Internal medicine	Physicians	Canada	Analytical cross-sectional studies
Ko et al, 2020^35^	Oncology	Physicians	Canada	Analytical cross-sectional studies
Kumar et al, 2020^36^	Outpatient oncology	Physicians, nurse practitioners, physician assistants	USA	Analytical cross-sectional studies and qualitative research
Kumar et al, 2023^37^	Oncology	Physicians, advance practice providers	USA	Quasi-experimental studies
Lagrotteria et al, 2021^38^	Tertiary hospitals	Physicians, nurse practitioners, social workers	Canada	Qualitative research
Lakin et al, 2019^39^	Primary care	Physicians, nurses, social workers	USA	Qualitative research
Lakin et al, 2021^40^	General medicine	Physicians, nurses, physician assistants	USA	Quasi-experimental studies
Lally et al, 2020^41^	Hospitalised patients in a complex care management program.	Nurses	USA	Quasi-experimental studies
Le et al, 2021^42^	Acute medicine	Physicians, nurses, allied health	Canada	Cohort studies
Locastro et al, 2023^43^	Hematology	Physicians, nurses, advance practitioners	USA	Qualitative research
Ma et al, 2020^44^	General internal medicine	Physicians, nurse practitioners	Canada	Quasi-experimental studies
Mandel et al, 2017^45^	Nephrology	Nephrologists, nurses, social workers, physicians	USA	Text and opinion
Mandel et al, 2023^46^	Dialysis	Physicians, social workers	USA	Qualitative research and analytical cross sectional studies
Massman et al, 2019^47^	Primary care	Physicians, physician assistants, nurses, medical assistants, social workers	USA	Quasi-experimental studies
McGlinchey et al, 2019^48^	U.K. healthcare setting	Clinicians—not specified	UK	Qualitative research
Miranda et al, 2018^49^	Oncology	Physicians, nurse practitioners	USA	Analytical cross sectional studies and qualitative research
Ouchi et al, 2020^50^	Emergency	Physicians	USA	Text and opinion
Paladino et al, 2019^51^	Oncology	Physicians, nurse practitioners, physician assistants	USA	Randomized controlled trials
Paladino et al, 2020^52^	Oncology	Physicians, advance practice clinicians	USA	Analytical cross sectional studies and qualitative research
Paladino et al, 2020^53^	Three health systems	Physicians, advance practice clinicians, nurses, social workers, chaplains	USA	Qualitative research and quasi-experimental studies
Paladino et al, 2021^54^	Acute and ambulatory care	Physicians, nurses, social workers	USA	Qualitative research
Paladino et al, 2021^55^	Primary care	Physicians, nurses, social workers	USA	Qualitative research
Paladino et al, 2022^56^	Three U.S. health systems	Physicians, advance practice clinicians, nurses, social workers, chaplains	USA	Qualitative research
Pasricha et al, 2020^57^	Intensive care	Physicians, nurses	USA	Analytical cross sectional studies and qualitative research
Rauch et al, 2023^58^	Education community health sites	Nursing students	USA	Qualitative research and quasi-experimental studies
Reed-Guy et al, 2021^59^	Glioblastoma	Physicians	USA	Analytical cross-sectional studies and qualitative research
Sanders et al, 2022^60^	Multiple contexts	Physicians	USA	Qualitative research and quasi-experimental studies
Sirianni et al, 2020^61^	COVID-19	Physicians	Canada	Text and opinion
Swiderski et al, 2021^62^	Primary care/community health	Physicians	USA	Qualitative research
Tam et al, 2019^63^	Family medicine, internal medicine	Medical students	Canada	Qualitative research and quasi-experimental studies
Thamcharoen et al, 2021^64^	Advanced kidney disease	Researcher	USA	Analytical cross-sectional studies and qualitative research
Van Breemen et al, 2020^65^	Pediatrics	Physicians, nurse practitioners, nurses, counselors	Canada	Case reports
Vergo et al, 2022^66^	Rural tertiary care center	Internal medicine residents	USA	Quasi-experimental studies
Wasp et al, 2021^67^	Hematology-Oncology	Oncology fellows	USA	Qualitative research and quasi-experimental studies
Xu et al, 2022^68^	Primary care	Physicians	USA	Qualitative research
Zehm et al, 2021^69^	Education	Medical students and interns	USA	Qualitative research and quasi-experimental studies

### Thematic Synthesis

Core competencies for SIC are described in 3 themes: (1) conversation resources, (2) intrapersonal capabilities, and (3) interpersonal capabilities. The themes describe *what* the competencies are, and the subthemes describe *how* the competencies are applied. For an overview of themes and subthemes, see Table 3.

**Table 3.** Overview of Core Competencies for SIC.

Themes	Subthemes
Conversation resources	Communicating with SICG as a tool
	Applying appropriate communication skills
Intrapersonal capabilities	Calibrating attitudes and mindset
	Feeling confident
Interpersonal capabilities	Building connectedness
	Demonstrating compassion
	Adjusting to preferences and conditions

SIC, serious illness conversation; SICG, Serious Illness Conversation Guide.

**Figure 2.**
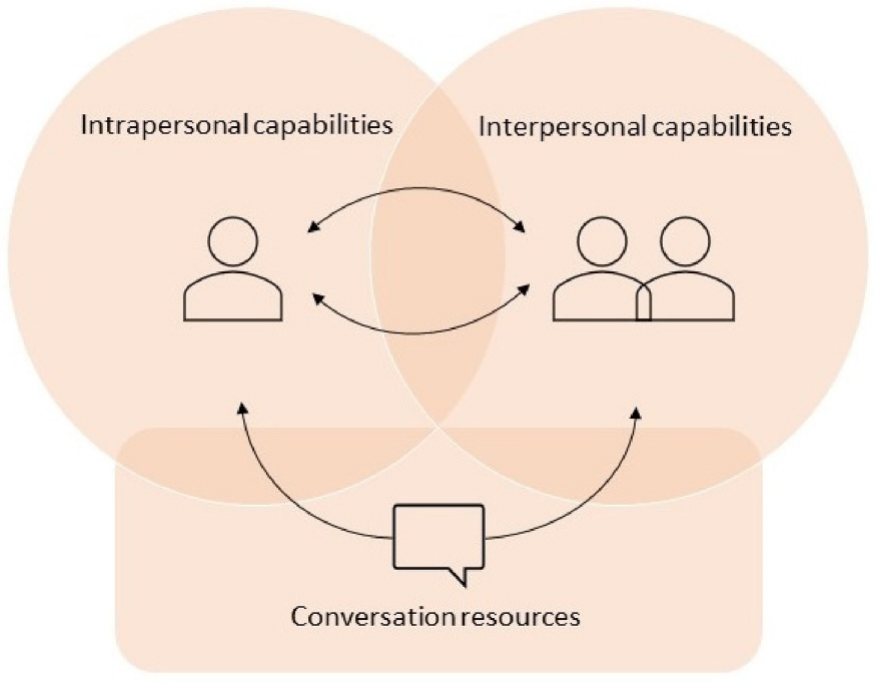
Conceptual model of core competencies for serious illness conversations.

Since competencies overlap and interlock, the themes and subthemes are not fully externally heterogeneous. Consequently, all competencies are important, and the clinician needs to be able to efficiently combine conversation strategies with intrapersonal and interpersonal skills to successfully conduct and interact in SIC. The 3 overarching core competencies and their relations are visualized in Figure 2. The arrows denote interactions between the 3 themes. The 2 arrows between the clinician—*intrapersonal capabilities*—and the meeting between clinician and patient—*interpersonal capabilities*—symbolize the circularity that exists between them. Meaning that intrapersonal capabilities can influence interpersonal capabilities and vice versa. Thus, having awareness of, and the ability to manage one's own attitudes and emotions can affect the success of the interpersonal aptitude of building connectedness, having an empathetic approach, and adapting the conditions to the patient as a person. Likewise, what happens in the interpersonal meeting influences the clinician's confidence and attitudes. The arrow going through *conversation resources* implies that efficient use of the SICG, together with applying appropriate communication skills, can influence the clinician's intrapersonal capability as well as enhance the success of the interpersonal meeting.

#### Conversation resources

Conversation resources included using the SICG as a tool while applying appropriate communication skills such as intentional silence, mirroring, and reflective questions. The content and structure of the SICG are followed in a flexible manner attuned to the clinician, patient, and family/caregivers.

##### Communicating with SICG as a tool

Using the guide effectively is a competence.^53^ The SICG was described as a tool that could be used to have a value-oriented^44,52^ active listening conversation^19^ by using the structure^12,20,24,26,39,53,64^ and asking the questions. Thus, the SICG offers a framework to structure SIC^53^ with concrete language^53^ and appropriate vocabulary.^53,63,69^ The guide demystified SIC^69^ and provided an agenda that went beyond mere conversations about highly medicalized end-of-life treatment preferences^38^ that is, focusing on goals instead of medical interventions.^39^ Optimal use of the guide^53^ included being able to elaborate upon the questions within it including enhancing their depth and breadth by asking questions such as “can you tell me more” and “what else.”^23^ This also included asking clarifying and follow-up questions,^19,20,23^ and utilizing examples if the patient does not grasp the question.^19^

To use the guide authentically, clinicians may need to adjust the guide to themselves^18^ as well as their patient population and culture.^18,45,63,68^ Clinicians should adapt the language to flow naturally in the conversation^48,55^ by including normalizing elements,^48,55^ and avoiding reading the guide verbatim or having an interview-like conversation.^48^ Adapting the guide to the patient required flexibility in thinking and operational style. This included language and format adjustments in accordance with patient needs^18^ and what was important to the patient.^48^ This means that clinicians should be able to incorporate the guide structure with patient responses.^23^ Flexibility was required in the application of the guide to respond to patient signals.^63^ “In the moment” use of the guide was described as desirable.^19,65^ The SICG could be used as a whole^65^ but should not be seen as fixed.^48^ Clinicians needed to choose when to use the guide questions^23,55^ and when to adjust the guide structure.^50,55^ Additionally, it was important to be ready to move “in” and “out” of the conversation to deal with present and urgent issues.^19^ Moreover, all guide elements did not need to be covered during a single conversation.^31^ Competence comes with practice, where the ability to actively listen without thinking about what to say^65^ and using the SICG “on the fly”^38^ advanced over time. Consequently, clinicians must find a balance between using the structure of the SICG and showing flexibility,^63^ for example by adjusting formulations to suit the patient.^45^ This is because people receive and share information in different ways and a one-size fits all framework may not function optimally.^24^ Taken together, SIC competencies ranged from a basic reading of the guide to more advanced competencies consisting of variation, clinical judgement, and creativity.^66^

##### Applying appropriate communication skills

Clinicians needed to be able to apply their unique communication skillset to appropriately navigate SIC.^31^ Deep^65^ and active listening were necessary competencies for SIC.^19,34,61,65,67^ Intentional silence^18^ could be utilized as a communication skill, where the clinician could choose when to respond directly and when to use the power of silence.^18,20,23,36,53^ Using silence allowed patients to process information,^20,40,54^ express emotions^20,26,40^ and thoughts.^26^ Speaking less than half of the time was a rule of thumb^20,26,30,36,40,53,61,63^ which created space for the patient's voice to be heard^20,54^ and for processing and reflection.^20,21,52,63,65^

Person-centered^54^ and gentle relatable language should be used^52^ that avoids medical jargon^31^ and lengthy monologues.^26^ Language and general tone could promote a supportive dialog^26^ which meant using neutral language^19^ and broader use of language—from medical to existential.^21^ Using open-ended questions was preferable^18,26,35,53,54^ since they empowered patients to share their stories.^54^ Additionally, it was important to create a respectful setting for SIC.^18^ Mirroring was a conversation skill that clinicians could apply to support patients to articulate and reflect upon their thoughts and emotions.^23^ Difficult emotions must be acknowledged.^40^ As such, emotion handling skills,^67,68^ including addressing and responding to emotions^18,20,23,26,36,45,53,63,65,68^ were essential. Embracing deep listening,^65^ avoiding looking away^36^ and using silence^53,54^ were nonverbal communication skills that could be used to respond to emotions. However, premature reassurance should be avoided and responding to emotions should be addressed by providing further *explanations*, rather than information alone.^20^

#### Intrapersonal capabilities

This theme included awareness of, and the ability to, interpret and calibrate the clinician's own attitudes and mindset to the SIC and the patient. Feeling confident comprised of self-assurance in engaging in SIC and included reflecting on clinicians’ comfort and possible discomfort.

##### Calibrating attitudes and mindset

The mindset and attitude of the clinician were connected to competence in having SIC.^53^ Shifting values in the context of SICP was achievable.^53^ Clinician attitudes toward SIC functioned both as an enabler and a barrier.^17^ This could be understood as having attitudes that either facilitated (ie, supported successful SIC) or constrained (ie, hindered successful SIC) competent engagement in SIC. Constraining attitudes could be reluctance to have SIC, concerns about the guide^53^ or causing suffering^17,53^ and anxiety,^56^ including fears of taking away hope.^17^ Furthermore, SIC could conflict with the professional role “to treat,” thereby hindering the adoption of SIC.^56^ On the other hand, acknowledging the value of SIC^53,63^ and viewing SIC as meaningful,^28,38,52^ favorable,^22^ important,^53^ humanizing,^38,62^ and supportive in times of crisis^54^ could be understood as facilitating attitudes (ie, supported successful mastery of SIC). Putting patient values in front of their own values^31^ and building facilitating attitudes was necessary.^53^ Additionally, attention to attitudes in relation to medical culture was needed.^27^ Shifting clinical values from a “medical focus” to a “person focus” with patient values at the forefront was possible.^38,53^

##### Feeling confident

The clinician needed to broach SIC with comfort and confidence.^30,40,53,55^ Having the SICG can support the clinician,^41,46,51,52^ however, the individual skills of the clinician were vital to its implementation.^48^ Confidence was required when soliciting values in relation to the patient's illness,^67^ feeling comfortable when exploring patient values and goals,^55^ and responding to emotions.^40^ Furthermore, it was important to feel confident in using silence,^40^ bringing up sensitive issues,^30^ such as trajectories, fears and wishes, as well as when conveying serious updates, managing conflicts, responding to unacceptance, and counseling patient requests.^42^ Uncertainty in how to express oneself^47,51,58^ and feeling discomfort^30^ were barriers to successful SIC.^30,47,51^ Consequently, the clinician needed to know how to act in challenging situations,^40^ such as when patients or family members expressed certain behaviors or emotions like crying, anger, denial, or avoidance.^20^ Clinician confidence and investment in SIC allowed them to take responsibility for these conversations.^31^ Discomfort and uncertainty could arise in the presence of strong emotions, low prognostic awareness by the patient, when delivering unwanted news and when worrying about upsetting the patient.^31^

The discomfort was especially described in relation to discussing prognosis.^23,27,33,55^ Balancing hope and reality could be challenging^59^ but it was vital for clinicians to have the capability to give honest statements^19^ on what could lie ahead.^59^ It was also important to acknowledge uncertainty and frame possibilities^45^ and discuss hope and positivity even in relation to poor prognosis.^43^ Recognizing reality while supporting hope was described as “gentle directness.”^26^ Clinicians portray this in practice by using the dual approach of “hoping for the best and preparing for the worst,”^35,45^ discussing SIC from a “hope and worry” perspective,^18,69^ or using the wish, worry, wonder framework.^65^

Becoming emotionally drained^26,37,56,62^ or feeling that engaging in SIC could bring back unpleasant memories from experiences of loss added another dimension to clinicians’ fears.^58^ Moreover, clinician comfort when approaching and discussing prognosis varied and was contingent on the role of the clinician, their experiences, culture, practice, and institution.^23^ Overall, reflecting on the root of one's discomfort in SIC was a vital competence,^31^ including the clinicians’ own cultural beliefs, personal biases, and possible stereotypes.^58^

#### Interpersonal capabilities

This theme focused on the clinician's ability to interact with the patient and family members to foster a mutually trusting relationship. It included empathetic communication, as well as attention and adherence to the situation and views of the patient and their family.

##### Building connectedness

Creating a sense of connection was necessary when developing therapeutic relationships in which skillful communication could occur.^19^ To build this connectedness in practice, the clinician needed to learn about the patient^35^ as a person^31^ and how to be engaged in a partnership.^45,48^ This included probing deeper to gain an understanding of what makes life meaningful for the patient.^61^ Furthermore, clinicians needed to provide space for family members to share their stories and understanding of the situation.^50^ Well-established relationships enhanced complex and emotional SIC,^24,39^ while poorly established relationships lowered trust.^39^ The importance of building rapport,^50,54,60^ clinician–patient alliance,^68^ relationships,^39,52,60,68^ closeness,^52^ warmth, and comfort^26^ were highlighted. The SICG could support connecting with patients,^60^ relationship development,^62^ building trust, and positive relationships.^57^ The clinician could create a sense of connection in the way that they enacted the conversation^19^ and by making it easier to have a challenging conversation by “smoothing the path.”^45^ Using “we” statements facilitated opportunities to develop a shared understanding.^24^

Building connection was furthermore linked to demonstrating strong rapport, which should be pursued early in the conversation.^54^ This could be built by referring to shared patient and clinician histories,^26,54^ using humor in a hard situation,^26^ and asking about and recognizing family members.^21,26^ Therapeutic alignment was central^31,32,38,50^ for building trust^50^ and signaled that patient's experience was essential to the therapeutic relationship.^32^ Moreover, attention to disparities in power dynamics between patient and clinician was required.^33^ Consequently, an open and equal dialogue should be pursued.^21^ Asking and seeking permission from patients and family members^18–20,24,31,35,45,50,54,63,65,66^ was another aspect of building connectedness. This included agreeing upon who should take part in the conversation,^45^ whether illness progression and prognosis should be discussed,^50,63^ and whether to proceed further with the discussion.^31,35,54^ Seeking permission fostered psychosocial safety for the patient^18^ and encouraged patients to maintain control over the discussion.^54^

##### Demonstrating Compassion

Demonstrating compassion included empathy and appropriate self-disclosure. Interpersonal competencies linked to empathy and self-disclosure encompassed being candid without being emotionally distant.^50,60^ This meant that the physician needed to get to know the patient on a personal level.^32^ Clinicians should take an empathetic approach to SIC^28,31,32^ with appropriate empathetic communication skills.^61^ In addition to verbal empathy, a demonstration of nonverbal empathy was needed.^18,35,54^ Empathetic nonverbal techniques included sitting down,^35^ being aware of one's facial expressions,^54^ making eye contact,^35^ and using therapeutic touch when appropriate.^54^ Responding to emotion using silence could likewise demonstrate nonverbal empathy.^18^ Demonstrating compassion when conducting SIC through telehealth could be challenging as it was not possible to see the whole person thus making it harder to respond to unspoken emotions and body language.^43^

Regarding verbal empathy, the wording in the SICG itself supported empathetic communication.^19,23,69^ Demonstrating verbal empathy included using caring language,^54^ understanding and naming emotions, stating respect, and offering support.^35^ Empathetic statements or actions should be used when responding to emotion,^20,31^ offering recommendations,^50^ and managing prognosis-related responses.^23^ Thus, empathetic and compassionate communication^54^ could support patient and family receptiveness and their ability to make choices that are best for them.^31^ The clinician should demonstrate non-abandonment^31^ and acknowledge the patient and family's emotions.^18,20,53^ Provision of reassurance was central,^26^ but should be authentic.^31^

##### Adapting to preferences and conditions

Adapting to preferences and conditions included trying to comprehend the patient from different viewpoints. This encompassed their holistic needs,^48^ including personal ambitions and goals,^25,45^ as well as their health-related goals, including care preferences^39,45^ and priorities.^59^ This also meant delving into patient concerns,^40^ including their personal and health-related fears and worries,^45^ by listening to patient expressions of illness understanding, losses, and uncertainties.^31^ Adapting to patient preferences and conditions required the cultivation of a deep and nuanced discussion,^49^ reflections,^19,50,63^ responding to expressed needs,^48,50^ and focusing on what was important to the patient.^21,22,26,32,39,52,61^

Adapting also included tailoring SIC to information preferences^18,26,35,39,45,48,52,53^ and making recommendations, planning and decisions with patient understanding, goals and values in mind.^26,32,38–40,45,48,50,52,65^ However, it was important to stress that it was not necessary to make decisions, solve problems, or reach conclusions about care.^19,26,45^ Moreover, clinicians must tailor the discussion to patient understanding,^52^ receptiveness,^20^ and readiness.^45,53,55^ At the same time, the role of the clinician also included guiding the patient's illness understanding^26^ by gently clarifying misunderstandings^19^ or unrealistic expectations,^57^ filling in knowledge gaps,^45^ and reframing patients’ expectations if necessary.^26^ Clinicians should be aware that patient preferences could vary depending on cultural beliefs,^31,42^ which required respect^31^ and culturally appropriate engagement in SIC^42^ to build cultural safety.^33^ Personal and societal characteristics and circumstances could vary, and clinicians should be mindful of language barriers,^34,62^ prior negative experiences of racism in healthcare, religions, health literacy, physical and cognitive ability, mental illness, poverty, or difficult family dynamics.^62^ It was therefore important for clinicians to be prepared to manage and adapt SIC according to patient and family contexts.^62^

## Discussion

This integrative systematic review identified core competencies for SIC. Skilled use and intuitive modification of conversation resources were necessary to meet the specific and individual needs of patients. This involved engaging intrapersonal capabilities within and around oneself, including tuning into attitudes and emotions and developing comfort and confidence discussing sensitive subjects. The findings emphasized that interpersonal capabilities that build and strengthen relationships and trust are foundational competencies for SIC.

Conversation guides and resources can act as a roadmap during important healthcare discussions to ensure that essential topics are addressed comprehensively. The structure of the SICG supports clinicians to focus on discussing patient values, goals and priorities, instead of focusing solely on medicine, procedures, and treatments.^12,70^ Patients experience satisfaction when reflecting on wishes and goals of care,^71^ and these discussions promote inclusion of people who are important to the patient, including their family, but also friends, caregivers, and others.^6^ One of the benefits of using a structured approach to clinical conversations is that standards of communication can be innovated, maintained, measured, and evaluated.^72^ This study revealed core competencies that describe not only “what” physicians do in SIC, but “how” they use and adapt structured conversation resources like the SICG. It is therefore important to further explore the balance between teaching a structured framework for these conversations while also promoting flexibility in clinicians’ approach to tailoring SIC for individual patients.

Advanced intra- and interpersonal communication skills can be enhanced through training to promote the integration of patient values and goals into treatment decisions.^73^ Training has been found to improve physicians’ understanding and expression of empathy and compassion by targeting specific behaviors and skills, such as detecting patients’ nonverbal emotional indicators, recognizing moments for compassion, conveyance of caring, and statements of validation and support.^74^ Despite training in SIC, Wasp et al^75^ concluded that physicians did not have full awareness of emotion regulation strategies in serious illness communication. Physicians described focusing on promoting reasonable hope in prognostic disclosure and building trusting and supportive relationships with patients. Yet, physicians’ own emotions in SIC were found to be variable and could conflict with their observed or expressed behavior.^75^ It seems clinician-learners could benefit from the opportunity to critically explore and identify their conscious and subconscious communication competencies, to develop what Lane and Roberts have termed “contextual reflective competence.”^76^ The findings from this study point toward the need for development of ongoing processes for reflection and support for clinicians to explore their beliefs, experiences and challenges in SIC as their intra- and interpersonal communication skills grow and develop.

SIC lives in the context of therapeutic alliance and relationships. Relational elements of communication are important and have a significant impact on building trust, improved adherence to treatment plans, and better overall health outcomes.^77^ Relationship-centered communication interventions have been linked to improved patient satisfaction and reduced clinician burnout.^78^ Patients and clinicians have both endorsed the importance of relationships in expressions and understandings of clinical empathy.^79^ A clinician who is relationship-oriented has been described as someone who “seeks to diminish the status gap in order to connect person-to-person with the patient”^79^ (Hall et al, p. 1240). Communication behaviors such as listening, understanding feelings and perspectives, and showing interest in the whole person are thought to augment patients’ experiences of empathy.^79^ The person-centered alliance that can be built in SIC may also be viewed from an equity standpoint, as literature shows that people from underrepresented or marginalized communities are less likely to receive positive nonverbal rapport-building communication and interactions that build trustworthiness.^80^ This is also important as it may diminish clinicians’ reliance solely on observed role modeling and self-practice to refine their capabilities.^81^ However, such interventions require support and commitment across all levels of the care continuum, inclusive of clinicians, leadership, and organizational structures.^82^ Developing a systems-level focus toward improving communication skills and delivering empathic and goal-concordant care may therefore better streamline and improve SIC experiences and outcomes.^83^

### Strengths and Limitations

A strength is that this review followed rigorous guidelines to identify relevant literature. Detailed descriptions of the search, selection, extraction, and analysis process have been provided and novel interpretations using thematic synthesis have been produced. There are several limitations to the review. This study only examined articles related to the SICP/SICG, so it is likely that these competencies will reflect some of the content from the program or guide. Competencies required for other serious illness communication training programs or guides (ie, not connected to Ariadne Labs’ SICP/SICG) were not explored as this was outside the study scope. It is possible that other care concepts and conversation models, for example, palliative care conversations and advanced care planning conversations, may cover related and/or comparable components. We suggest that future studies build upon these results to compare the conceptual and conversational aspects in relation to clinician competence. Lastly, 4 authors in the current study authored several included articles (SA (*n* = 1), JP (*n* = 16), EKF (*n* = 7), AS (*n* = 1)). To minimize the risk of bias, these authors were not involved in the article selection, data extraction or quality appraisal processes.

## Conclusions

Core competencies for SIC encompass a combination of conversation resources, intrapersonal capabilities, and interpersonal capabilities. Clinicians’ aptitude in communication, relationship-building, self-efficacy, and use of resources impact their competence to undertake SIC. Future training in SIC could focus on enhancing these areas to improve the quality of these conversations for patients and clinicians.

## Supplemental Material

sj-docx-1-pal-10.1177_08258597241245022 - Supplemental material for Core Competencies for Serious Illness Conversations: An Integrative Systematic Review Supplemental material, sj-docx-1-pal-10.1177_08258597241245022 for Core Competencies for Serious Illness Conversations: An Integrative Systematic Review by Susanna Pusa, Rebecca Baxter, Sofia Andersson and Erik K. Fromme, Joanna Paladino, Anna Sandgren in Journal of Palliative Care
