# Supplementary material for: Core Competencies for Serious Illness Conversations: An Integrative Systematic Review
Source: J Palliat Care. 2024 Apr 1;39(4):340–51. doi: 10.1177/08258597241245022 (PMC11528878; doi:10.1177/08258597241245022)
Supplement: sj-docx-1-pal-10.1177_08258597241245022 - Supplemental material for Core Competencies for Serious Illness Conversations: An Integrative Systematic Review [file sj-docx-1-pal-10.1177_08258597241245022.docx]

**Supplementary Material A**

**Search strategy**

| Database | Search strategy | Records retrieved |
| --- | --- | --- |
| CINAHL | AB “serious illness communication” OR AB “serious illness program*” OR AB “serious illness care” OR AB “serious illness conversation*” OR AB “serious illness model”  Limited to English, publications from 2014-01, academic journals. | 148 |
| MEDLINE | AB “serious illness communication” OR AB “serious illness program*” OR AB “serious illness care” OR AB “serious illness conversation*” OR AB “serious illness model”  Limited to English, publications from 2014-01, academic journals. | 211 |
| PsychInfo | AB “serious illness communication” OR “AB serious illness program*” OR AB “serious illness care” OR AB “serious illness conversation*” OR AB “serious illness model”  Limited to English, publications from 2014, academic journals. | 60 |
| PubMed | ("serious illness communication"[Title/Abstract]) OR ((((serious illness program*[Title/Abstract]) OR (serious illness care[Title/Abstract])) OR (serious illness conversation*[Title/Abstract])) OR (serious illness model[Title/Abstract]))  Limited to English, publications from 2014-01-01. | 279 |

**Supplementary Material B**

**JBI Quality Appraisal Checklists**

| **Checklist for Randomized Controlled Trials** | | | | | | | | | | | | | |
| --- | --- | --- | --- | --- | --- | --- | --- | --- | --- | --- | --- | --- | --- |
| **Articles** | **Q1** | **Q2** | **Q3** | **Q4** | **Q5** | **Q6** | **Q7** | **Q8** | **Q9** | **Q10** | **Q11** | **Q12** | **Q13** |
| Paladino et al. ^(51)^ | Y | Y | Y | Y | N | Y | Y | Y | Y | Y | Y | U | Y |

^Y = Yes; N= No; U = Unsure; N/A = Not applicable.^
*Tufanaru C, Munn Z, Aromataris E, Campbell J, Hopp L. Chapter 3: Systematic reviews of effectiveness. In: Aromataris E, Munn Z (Editors)*. JBI Manual for Evidence Synthesis. JBI, 2020. Available from https://synthesismanual.jbi.global

| **Checklist for Quasi-Experimental Studies** | | | | | | | | | |
| --- | --- | --- | --- | --- | --- | --- | --- | --- | --- |
| **Articles** | **Q1** | **Q2** | **Q3** | **Q4** | **Q5** | **Q6** | **Q7** | **Q8** | **Q9** |
| Lakin et al. ^(40)^ | Y | N | Y | Y | N | Y | Y | Y | Y |
| Wasp et al.* ^(67)^ | U | Y | U | N | N | N/A | Y | U | U |
| Ma et al. ^(44)^ | Y | Y | Y | N | N | N/A | Y | Y | U |
| Paladino et al.* ^(53)^ | N | Y | N | N | N | N/A | Y | U | Y |
| Massman et al. ^(47)^ | U | Y | U | N | N | N/A | Y | U | U |
| Tam et al.* ^(63)^ | Y | Y | Y | N | N | N/A | Y | U | Y |
| Lally et al. ^(41)^ | N | U | Y | N | N | N/A | Y | U | U |
| Zehm et al.* ^(69)^ | U | Y | U | N | N | N/A | Y | U | U |
| Hafid et al.* ^(30)^ | U | Y | Y | N | N | N/A | Y | U | U |
| Sanders et al.* ^(60)^ | U | Y | U | N | N | N/A | Y | U | U |
| Rauch et al.* ^(58)^ | U | Y | U | N | N | N/A | Y | U | U |
| Kumar et al. ^(37)^ | U | N | U | N | N | N/A | Y | U | U |
| Vergo et al. ^(66)^ | U | Y | U | N | N | N/A | Y | U | U |

^Y = Yes; N= No; U = Unclear; N/A = Not applicable; * = mixed method/multiple checklists.^*Tufanaru C, Munn Z, Aromataris E, Campbell J, Hopp L. Chapter 3: Systematic reviews of effectiveness. In: Aromataris E, Munn Z (Editors).* JBI Manual for Evidence Synthesis. JBI, 2020. Available from https://synthesismanual.jbi.global

| **Checklist for Cohort Studies** | | | | | | | | | | | |
| --- | --- | --- | --- | --- | --- | --- | --- | --- | --- | --- | --- |
| **Articles** | **Q1** | **Q2** | **Q3** | **Q4** | **Q5** | **Q6** | **Q7** | **Q8** | **Q9** | **Q10** | **Q11** |
| Le et al. ^(42)^ | Y | Y | Y | Y | U | U | Y | Y | U | Y | Y |
| Greenwald et al. ^(28)^ | Y | Y | U | Y | U | U | U | Y | Y | Y | Y |
| Gace et al. ^(25)^ | U | U | U | Y | U | U | Y | U | N | N | Y |

^Y = Yes; N= No; U = Unclear; N/A = Not applicable.^*Moola S, Munn Z, Tufanaru C, Aromataris E, Sears K, Sfetcu R, Currie M, Qureshi R, Mattis P, Lisy K, Mu P-F. Chapter 7: Systematic reviews of etiology and risk . In: Aromataris E, Munn Z (Editors)*. JBI Manual for Evidence Synthesis. JBI, 2020. Available from https://synthesismanual.jbi.global

| **Checklist for Analytical Cross Sectional Studies** | | | | | | | | |
| --- | --- | --- | --- | --- | --- | --- | --- | --- |
| **Articles** | **Q1** | **Q2** | **Q3** | **Q4** | **Q5** | **Q6** | **Q7** | **Q8** |
| Thamcharoen et al.* ^(64)^ | Y | Y | Y | Y | U | U | U | Y |
| Greenwald et al. ^(29)^ | Y | Y | U | U | N | N | U | Y |
| Kumar et al.* ^(36)^ | Y | Y | Y | Y | Y | N | U | Y |
| Geerse et al.* ^(27)^ | Y | Y | U | U | Y | U | U | Y |
| Paladino et al.* ^(52)^ | Y | Y | U | U | Y | N | U | Y |
| Miranda et al.* ^(49)^ | Y | Y | U | Y | Y | N | U | Y |
| Daubman et al. ^(23)^ | Y | Y | U | U | N | N | U | U |
| Ko et al. ^(35)^ | N | N | Y | U | Y | Y | U | Y |
| Pasricha et al.* ^(57)^ | Y | Y | U | Y | Y | N | U | Y |
| Reed-Guy et al.* ^(59)^ | Y | Y | U | Y | Y | N | U | Y |
| Daly et al. ^(22)^ | Y | Y | U | U | N | N | U | Y |
| King et al. ^(34)^ | Y | Y | Y | Y | Y | Y | U | Y |
| Mandel et al. * ^(46)^ | Y | Y | U | Y | U | N | U | Y |

^Y = Yes; N= No; U = Unclear; N/A = Not applicable; * = mixed method/multiple checklists.^*Moola S, Munn Z, Tufanaru C, Aromataris E, Sears K, Sfetcu R, Currie M, Qureshi R, Mattis P, Lisy K, Mu P-F. Chapter 7: Systematic reviews of etiology and risk . In: Aromataris E, Munn Z (Editors)*. JBI Manual for Evidence Synthesis. *JBI, 2020. Available from https://synthesismanual.jbi.global*

| **Checklist for Qualitative Research** | | | | | | | | | | |
| --- | --- | --- | --- | --- | --- | --- | --- | --- | --- | --- |
| **Articles** | **Q1** | **Q2** | **Q3** | **Q4** | **Q5** | **Q6** | **Q7** | **Q8** | **Q9** | **Q10** |
| Thamcharoen et al * ^(64)^ | Y | Y | Y | Y | Y | Y | U | Y | Y | Y |
| Paladino et al. ^(54)^ | Y | Y | U | U | U | Y | N | U | N | U |
| DeCourcey et al. ^(24)^ | Y | Y | Y | Y | Y | Y | Y | Y | N | Y |
| Paladino et al. ^(55)^ | Y | Y | Y | Y | Y | Y | U | Y | Y | Y |
| Kumar et al.* ^(36)^ | Y | Y | Y | Y | Y | N | U | Y | Y | Y |
| Geerse et al.* ^(27)^ | U | Y | Y | U | U | N | U | U | Y | Y |
| Wasp et al.* ^(67)^ | U | U | Y | Y | Y | N | N | Y | Y | U |
| Paladino et al.* ^(52)^ | Y | Y | Y | Y | Y | N | U | Y | Y | Y |
| Paladino et al.* ^(53)^ | Y | Y | Y | Y | Y | N | U | Y | Y | Y |
| Tam et al.* ^(63)^ | U | U | Y | U | Y | N | N | Y | Y | Y |
| McGlinchey et al. ^(48)^ | Y | Y | Y | Y | Y | N | N | Y | Y | Y |
| Geerse et al. ^(26)^ | Y | Y | Y | Y | Y | Y | Y | Y | Y | Y |
| Lakin et al. ^(39)^ | Y | Y | Y | Y | Y | Y | Y | Y | Y | Y |
| Miranda et al.* ^(49)^ | U | U | U | U | U | N | N | Y | Y | U |
| Pasricha et al.* ^(57)^ | U | Y | U | U | U | N | U | Y | Y | Y |
| Zehm et al.* ^(69)^ | U | U | Y | U | U | N | N | U | N | Y |
| Borregaard Myrhøj et al. ^(21)^ | Y | Y | Y | Y | Y | Y | Y | Y | N | Y |
| Reed-Guy et al.* ^(59)^ | Y | Y | Y | U | Y | N | N | Y | Y | Y |
| Lagrotteria et al. ^(38)^ | Y | Y | Y | Y | Y | Y | Y | Y | Y | Y |
| Hafid et al.* ^(30)^ | Y | Y | Y | U | U | Y | N | U | Y | Y |
| Swiderski et al. ^(62)^ | Y | Y | Y | Y | Y | Y | Y | Y | N | Y |
| LoCastro et al. ^(43)^ | U | Y | Y | Y | Y | N | U | Y | Y | Y |
| Sanders et al.* ^(60)^ | U | Y | Y | Y | Y | U | U | Y | Y | Y |
| Xu et al. ^(68)^ | U | Y | Y | Y | Y | N | U | Y | Y | Y |
| Andersson et al. ^(17)^ | Y | Y | Y | Y | Y | N | U | Y | Y | Y |
| Paladino et al. ^(56)^ | Y | Y | Y | Y | Y | Y | Y | Y | Y | Y |
| Rauch et al.* ^(58)^ | U | Y | Y | U | Y | N | N | Y | Y | Y |
| Mandel et al.* ^(46)^ | U | Y | Y | Y | Y | N | U | Y | Y | Y |

^Y = Yes; N= No; U = Unclear; N/A = Not applicable; * = mixed method/multiple checklists.^*Lockwood C, Munn Z, Porritt K. Qualitative research synthesis: methodological guidance for systematic reviewers utilizing meta-aggregation. Int J Evid Based Healthc. 2015;13(3):179–187.*

| **Checklist for Case Reports** | | | | | | | | |
| --- | --- | --- | --- | --- | --- | --- | --- | --- |
| **Articles** | **Q1** | **Q2** | **Q3** | **Q4** | **Q5** | **Q6** | **Q7** | **Q8** |
| Van Breemen et al. ^(65)^ | Y | Y | Y | Y | Y | Y | Y | Y |

^Y = Yes; N= No; U = Unclear; N/A = Not applicable.^
*Moola S, Munn Z, Tufanaru C, Aromataris E, Sears K, Sfetcu R, Currie M, Qureshi R, Mattis P, Lisy K, Mu P-F. Chapter 7: Systematic reviews of etiology and risk. In: Aromataris E, Munn Z (Editors)*. JBI Manual for Evidence Synthesis. JBI, 2020. Available from https://synthesismanual.jbi.global

| **Checklist for Text and Opinion** | | | | | | |
| --- | --- | --- | --- | --- | --- | --- |
| **Articles** | **Q1** | **Q2** | **Q3** | **Q4** | **Q5** | **Q6** |
| Beddard-Huber et al. ^(19)^ | Y | Y | Y | Y | Y | Y |
| Sirianni et al. ^(61)^ | Y | Y | Y | Y | Y | Y |
| Jain et al. ^(31)^ | Y | Y | Y | Y | Y | Y |
| Baran et al. ^(18)^ | Y | Y | Y | Y | Y | Y |
| Mandel et al. ^(45)^ | Y | Y | Y | Y | Y | Y |
| Bernacki et al. ^(20)^ | Y | Y | Y | Y | Y | Y |
| Ouchi et al. ^(50)^ | Y | Y | Y | Y | Y | Y |
| Karim et al. ^(33)^ | Y | Y | Y | Y | Y | Y |
| Jacobsen et al. ^(32)^ | Y | Y | Y | Y | Y | Y |

^Y = Yes; N= No; U = Unsure; N/A = Not applicable.^
*McArthur A, Klugarova J, Yan H, Florescu S. Innovations in the systematic review of text and opinion. Int J Evid Based Healthc. 2015;13(3):188–195.*
